# Exploring the molecular mechanisms of huperzine a in the treatment of rosacea through network pharmacology, machine learning, and molecular dynamics simulations

**DOI:** 10.3389/fphar.2025.1586829

**Published:** 2025-05-22

**Authors:** Xin Luo, Suhan Yang, Lian Zhong, Peng Zhang

**Affiliations:** ^1^ Department of Dermatology, Second Xiangya Hospital, Hunan Key Laboratory of Medical Epigenomics, Clinical Medical Research Center of Major Skin Diseases and Skin Health of Hunan Province, Central South University, Changsha, Hunan, China; ^2^ Department of Blood Transfusion, Pingxiang People’s Hospital, Gannan Medical University, Pingxiang, China

**Keywords:** rosacea, huperzine A, machine learning, network pharmacology, molecular dynamics simulation

## Abstract

**Introduction:**

Rosacea is a common chronic inflammatory skin disorder and dysregulation of neuroimmune functions and neurovascular loops play critical roles in the development of rosacea. Huperzine A (Hup A) has several bioactive properties, including anti-inflammatory, antioxidant, and neuroprotective effects. However, the potential roles of Hup A in treating rosacea is unknown.

**Methods:**

Network pharmacology, molecular docking, and molecular dynamics simulation techniques has been used to investigate the anti-rosacea mechanisms of Hup A in rosacea.

**Results:**

Our results predicted 21 potential anti-rosacea targets of Hup A through public databases. KEGG pathway enrichment analysis revealed that these key targets participated in the regulation of MAPK signaling, NF-kappa B signaling, and PI3KAKT signaling pathways. Further machine learning analysis identified six core targets (BCL2, RXRA, PKN2, XDH, PRKCA, and FAP). Analysis of the GSE65914 dataset showed that XDH was upregulated in rosacea lesions, while BCL2 and RXRA were downregulated, with no significant expression changes of the other genes. Molecular docking results indicated that Hup A could bind to key targets (XDH, BCL2, and RXRA), which were further confirmed by molecular dynamics simulations.

**Discussion:**

This study systematically elucidates the potential mechanisms of Hup A in the treatment of rosacea and provides a theoretical basis for its application in rosacea therapy.

## 1 Introduction

Rosacea is a persistent and recurring inflammatory skin disorder that predominantly impacts the prominent facial regions, such as the cheeks, nose, forehead, and chin. It is distinguished by erythema, which may be transient or chronic, along with telangiectasia and inflammatory lesions that include papulo-pustules and edema (Sharma et al., 2022). The global prevalence of rosacea is estimated to be 5.46% (Gether et al., 2018). The exact mechanisms of rosacea remain unknown, involving the contributions of both genetic and environmental influences. Exposure to sunlight, heat or cold, alcohol, spicy foods, or exercising are the trigger factors of rosacea, which can activate peripheral sensory nerve endings and contribute to the development of rosacea (Steinhoff et al., 2011; Steinhoff and Bergstresser, 2011). Rosacea has been reported to be associated with metabolic, psychiatric, and neurological disorders, including Alzheimer’s disease (AD) (Chen et al., 2020) and Parkinson’s disease (PD) (Egeberg et al., 2016). Reports have indicated that anti-depressants like mirtazapine and paroxetine can modulate neurovascular function and neuroinflammation, providing therapeutic benefits for rosacea (Waldinger et al., 2000; Wang B. et al., 2023). Gabapentin, an anticonvulsant, has also been shown to significantly alleviate skin erythema in patients with rosacea (Ma et al., 2024). Therefore, the dysregulation of mediators and receptors involved in neurovascular and neuroimmune interactions may play a pivotal role in the initial phases of rosacea. Therapeutic agents targeting neurovascular and/or neuroimmune pathways could prove advantageous in managing rosacea (Schwab et al., 2011).

Huperzine A (Hup A), an alkaloid extracted from Huperzia serrata, was known for its neuroprotective, anti-inflammatory, and immune-modulating effects (Mei et al., 2017). Hup A has been used to treat AD, schizophrenia, and memory impairments (Tsai, 2019; Zhang et al., 2007; Ha et al., 2011; Khanal et al., 2021). Additionally, Hup A has been shown to effectively improve cognitive dysfunction and reduce neuroinflammation. In the N-methyl-d-aspartate (NMDA) -induced seizure rat models, Hup A protects against seizure and status epilepticus by blocking NMDA-induced excitotoxicity *in vivo* (Coleman et al., 2008). In a 1-methyl-4-phenyl-1,2,3,6-tetrahydropyridine (MPTP)-induced PD mouse model, Hup A significantly relieved motor behavior and cognitive functions and prevented the degeneration of dopaminergic neurons (Guo et al., 2023). Recent findings have revealed that Hup A can enhance cognitive functions related to learning and memory in both animal models and patients with AD (Zhang and Tang, 2006). In summary, Hup A appears to be a potential therapeutic agent for rosacea.

Although Hup A exhibits multiple pharmacological properties, including anti-inflammatory, antioxidant, and neuroprotective effects, its role in the treatment of rosacea remains unclear. To gain deeper insights into the pharmacological mechanisms underlying drug actions in disease treatment, researchers have increasingly employed computational approaches such as network pharmacology, molecular docking, and multidimensional bioinformatics analyses to investigate disease pathogenesis and therapeutic mechanisms (Wei et al., 2024; Li et al., 2022; Wang L. et al., 2023).

This study aimed to comprehensively elucidate the anti-rosacea potential of Hup A, identify its therapeutic targets, and explore the underlying mechanisms through network pharmacology, molecular docking, and molecular dynamics simulations. The findings of this study provide a theoretical foundation for the further application of HupA in the treatment of rosacea.

## 2 Methods

### 2.1 Hup A target identification

The SMILES identifier of Hup A was retrieved from the PubChem database (https://pubchem.ncbi.nlm.nih.gov/). Putative targets of Hup A were systematically predicted using three independent platforms: the SuperPred database, SwissTargetPrediction database (http://www.swisstargetprediction.ch/), and Comparative Toxicogenomics Database (https://ctdbase.org/). Predicted targets from these databases were consolidated, and duplicate entries were removed through rigorous deduplication procedures. This integrative approach yielded 222 unique molecular targets associated with Hup A.

### 2.2 Prediction of rosacea-related target genes

Rosacea-associated targets were systematically identified from the DisGeNET (https://www.disgenet.org/) and GeneCards (https://www.genecards.org/) databases using the search term “rosacea.” Candidate targets from both repositories were merged, and redundant entries were eliminated through computational deduplication. This integrated curation process yielded 632 non-redundant molecular targets linked to rosacea pathogenesis.

### 2.3 Identification of common drug - disease targets and protein - protein interaction network construction

To identify potential therapeutic targets of Hup A for rosacea, intersecting targets between rosacea-associated genes and Hup A targets were analyzed using the Jvenn online platform (http://www.bioinformatics.com.cn/static/others/jvenn/example.html) to generate a Venn diagram. The 21 shared targets were subsequently imported into the STRING database (http://string-db.org/) for protein-protein interaction (PPI) network construction. The PPI network was further visualized using Cytoscape version 3.9.1, and the degree value of each target was calculated using the NetworkAnalyzer plugin.

### 2.4 KEGG enrichment analysis

KEGG pathway enrichment analysis of the 21 shared targets was performed using the DAVID bioinformatics platform (https://david.ncifcrf.gov/). Enrichment results were visualized through the Sangerbox integrated analysis tool kit (http://www.sangerbox.com/).

### 2.5 Machine learning-based Identification of key targets

Three machine learning algorithms were employed to identify core targets. 1) LASSO Regression: Implemented via the R glmnet package with 3-fold cross-validation for feature selection. 2) SVM-RFE: A supervised classification algorithm combining support vector machines (SVM) with recursive feature elimination (RFE) to optimize variable combinations. 3) Random Forest: Constructed using the R randomForest package, with cross-validated feature importance ranking. Consensus targets identified by all three algorithms were defined as core therapeutic targets.

### 2.6 Evaluation of key targets expression and diagnostic efficacy

The mRNA expression levels of six core targets in rosacea lesions *versus* normal skin were analyzed using the GSE65941 dataset. Receiver operating characteristic (ROC) curves were generated, with area under the curve (AUC) values calculated to evaluate diagnostic potential.

### 2.7 Immune infiltration analysis

CIBERSORT deconvolution analysis (1,000 permutations, p < 0.05) quantified immune cell infiltration differences between rosacea lesions and normal skin in GSE65941. Visualization was achieved using: Stacked bar plots for immune cell proportions, Heatmaps (R pheatmap package) for 22 immune cell types, Violin plots (R vioplot package) for abundance comparisons. Correlation heatmaps (R corrplot) revealed associations between immune cells and core target expression.

### 2.8 Molecular docking

Hup A’s 2D structure (PubChem: http://pubchem.ncbi.nlm.nih.gov/) was converted to an energy-minimized 3D mol2 file using ChemOffice. Target protein structures (PDB: http://www.rcsb.org/) were prepared via PyMOL (removal of water/phosphates) and AutoDock4 (hydrogen addition). Molecular docking was performed in MOE 2019, with binding affinity evaluated by free energy. Results were visualized using PyMOL and Discovery Studio.

### 2.9 Molecular dynamics simulation

Hup A-XDH complex interactions were simulated using GROMACS 2020.6 under AMBER99SB force field and SPC water model (300 K, 100 ns). System preparation included: Energy minimization via steepest descent algorithm, Equilibration phases (NVT/NPT ensembles), Production MD simulations. Trajectory analysis and visualization were performed using Xmgrace 5.1.25.

## 3 Results

### 3.1 Identification of potential targets of Hup A in the treatment of rosacea

**FIGURE 1:**
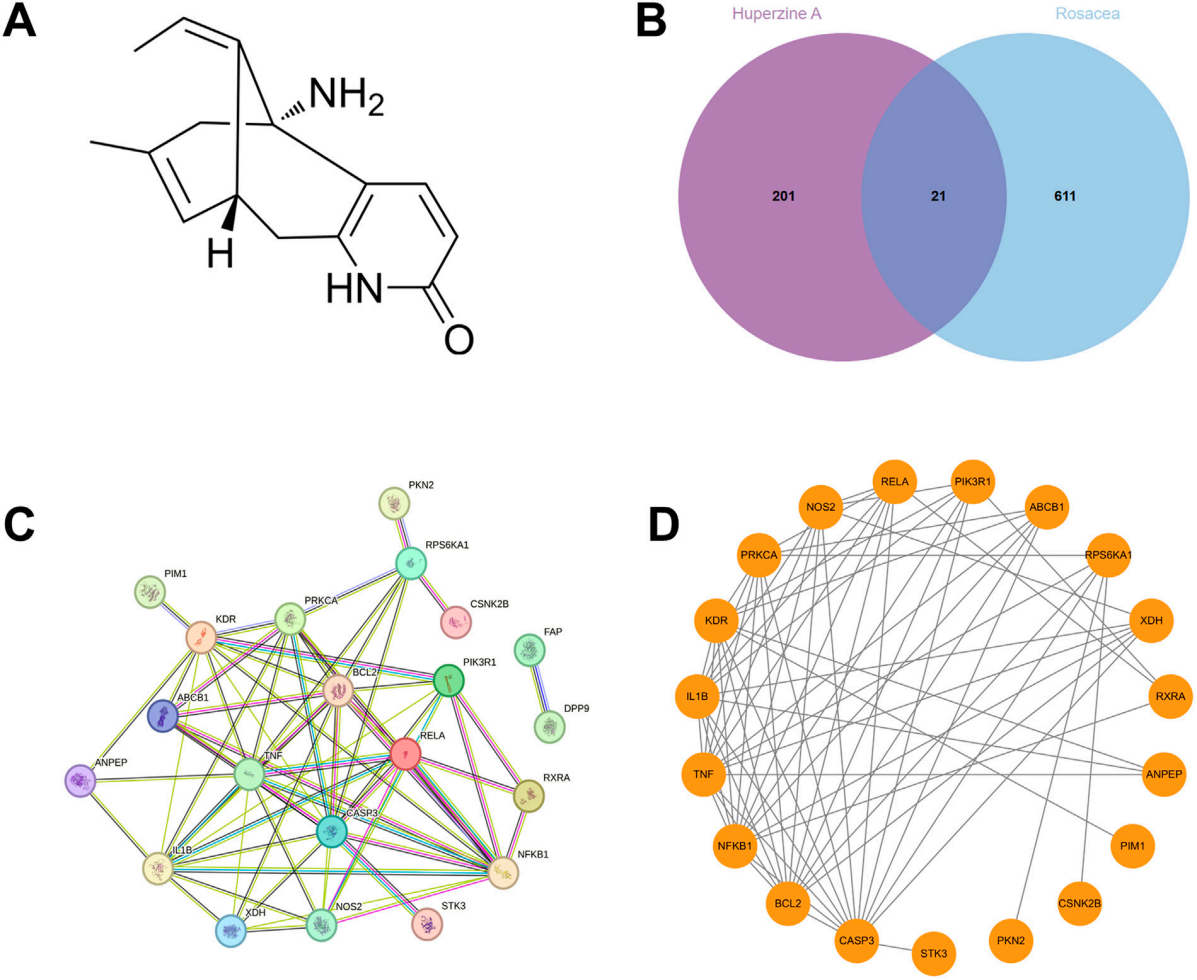
Identification of potential targets of Hup A in the treatment of rosacea by network pharmacology analysis. **(A)** Structural formulas of Hup A. **(B)** Candidate targets (n = 222) were identified through PharmMapper, SwissTargetPrediction, and SuperPred databases. Rosacea-associated targets (n = 632) were retrieved from GeneCards and DisGeNET. And 21 target genes were overlapped the between Hup A-related genes and those associated with rosacea. **(C,D)** Protein–protein interaction (PPI) network of 21 intersecting targets.

Network pharmacology analysis was conducted to investigate the therapeutic potential role of Hup A in rosacea. Based on Hup A’s chemical structure (Figure 1A), 222 candidate targets were identified through PharmMapper, SwissTargetPrediction, and SuperPred databases. Rosacea-associated targets (n = 632) were retrieved from GeneCards and DisGeNET. Intersection analysis via Venn diagram (Figure 1B) revealed 21 shared targets.

PPI network of the 21 overlapping targets was constructed using the STRING database, comprising 21 nodes and 66 edges, with an average node degree of 6.29 (Figure 1C). As FAP and DPP9 were isolated from the main network with a degree of only 1, they were excluded from further analysis. The remaining network, consisting of 19 nodes and 65 edges, was subsequently visualized using Cytoscape version 3.9.1 (Figure 1D). The refined network exhibited an average node degree of 6.84, with node degree values summarized in Table 1.

**TABLE 1:** The node degree values from PPI.

Gene	Degree unDir
CASP3	13
NFKB1	12
BCL2	12
TNF	12
IL1B	11
KDR	10
PRKCA	9
NOS2	8
RELA	8
PIK3R1	7
ABCB1	6
XDH	6
RPS6KA1	6
ANPEP	3
RXRA	3
STK3	1
CSNK2B	1
PIM1	1
PKN2	1

### 3.2 KEGG enrichment analysis

**FIGURE 2:**
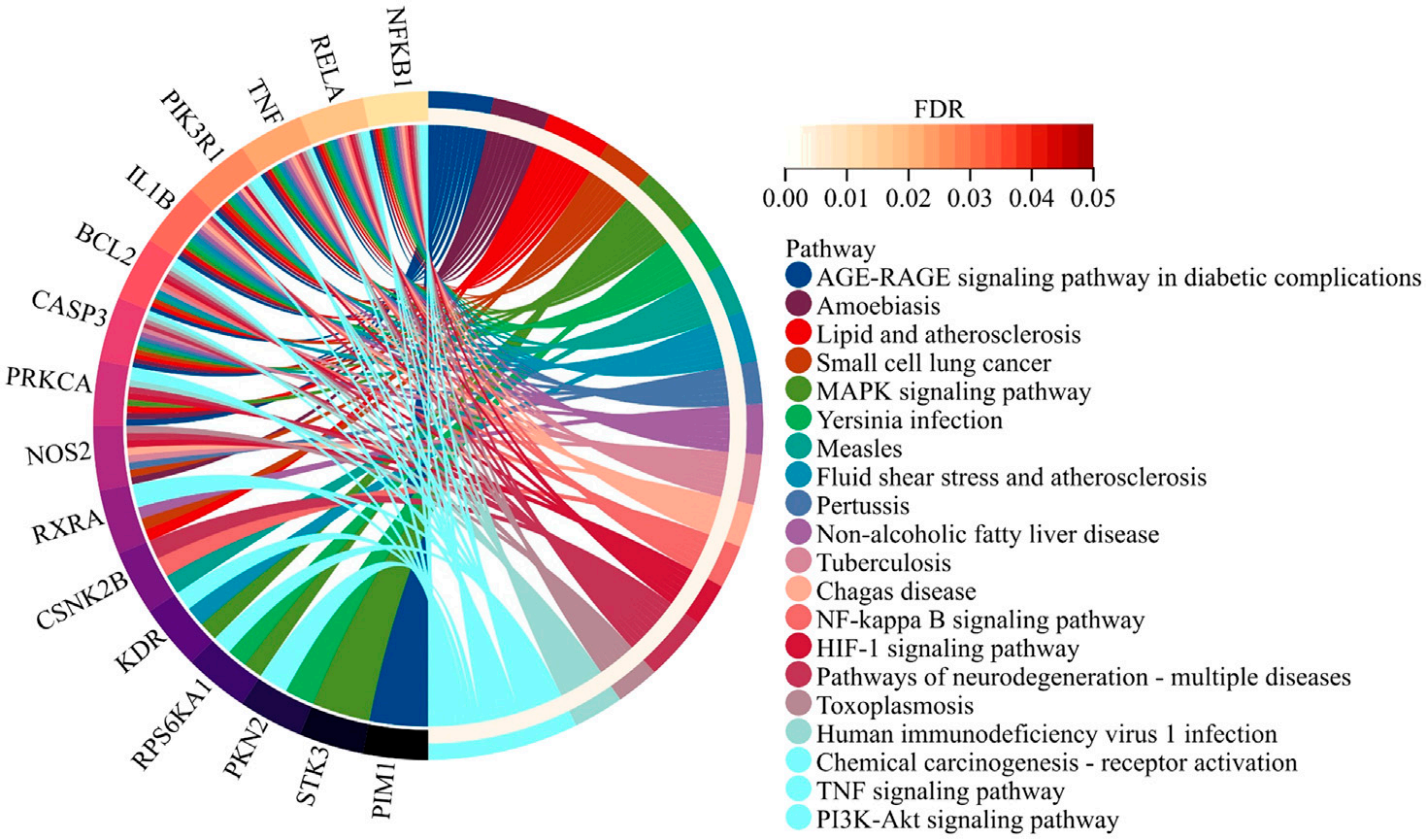
KEGG pathway enrichment analysis. The analysis of 21 overlapping genes revealed that Hup A primarily modulates the MAPK signaling pathway, NF-κB signaling pathway, and PI3K-AKT signaling pathway in rosacea treatment.

KEGG pathway enrichment analysis of the 21 overlapping genes was performed to preliminarily delineate the mechanistic basis of Hup A’s therapeutic effects through network pharmacology. Key targets mediating the interaction between Hup A and rosacea were further analyzed via KEGG enrichment using the DAVID database, aiming to clarify the potential roles of candidate targets. The analysis revealed that Hup A primarily modulates the MAPK signaling pathway, NF-κB signaling pathway, and PI3K-AKT signaling pathway in rosacea treatment (Figure 2). These pathways are critically associated with inflammatory regulation and cellular survival, supporting Hup A’s mechanistic relevance in targeting rosacea pathogenesis.

### 3.3 Six DEGs identified as specific targets of Hup A in the treatment of rosacea

**FIGURE 3:**
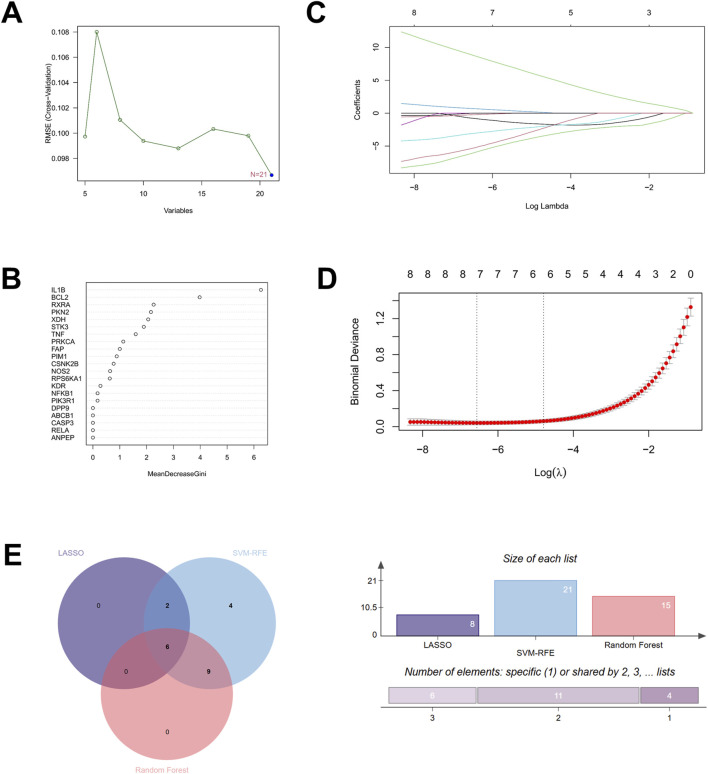
Machine learning algorithms identified six core therapeutic targets of Hup A for the treatment of rosacea. **(A)** SVM-RFE feature curve **(B)** Random forest algorithm **(C,D)** LASSO regression model **(E)** Venn diagram showing the overlap between SVM-RFE, Random forest and LASSO algorithms, identifying common core targets for Hup A-induced rosacea.

To further screen potential core therapeutic targets of Hup A in the treatment of rosacea, we employed three machine learning algorithms: SVM-RFE, random forest, and LASSO to identify key targets among the 21 candidate therapeutic targets. First, using the SVM-RFE algorithm with radial basis kernel functions, we performed cross-validated feature selection on gene expression data of candidate targets through the rfe function. The relationship between feature numbers and model RMSE (root mean square error) was plotted for evaluation, revealing that all 21 therapeutic targets met the criteria for optimal targets (Figure 3A). Subsequently, random forest algorithm was applied to rank target importance scores, yielding 15 optimal therapeutic targets (Figure 3B). Then, LASSO logistic regression with three-fold cross-validated penalty parameter adjustment identified 8 genes as potential core therapeutic targets (Figures 3C,D). Finally, a Venn diagram analysis intersecting core genes identified by these three machine learning approaches revealed six consensus core targets: BCL2, retinoid X receptor alpha (RXRA), PKN2, Xanthine dehydrogenase (XDH), PRKCA, and FAP (Figure 3E).

### 3.4 Validation of the expression and diagnostic efficacy of six core targets in rosacea

**FIGURE 4:**
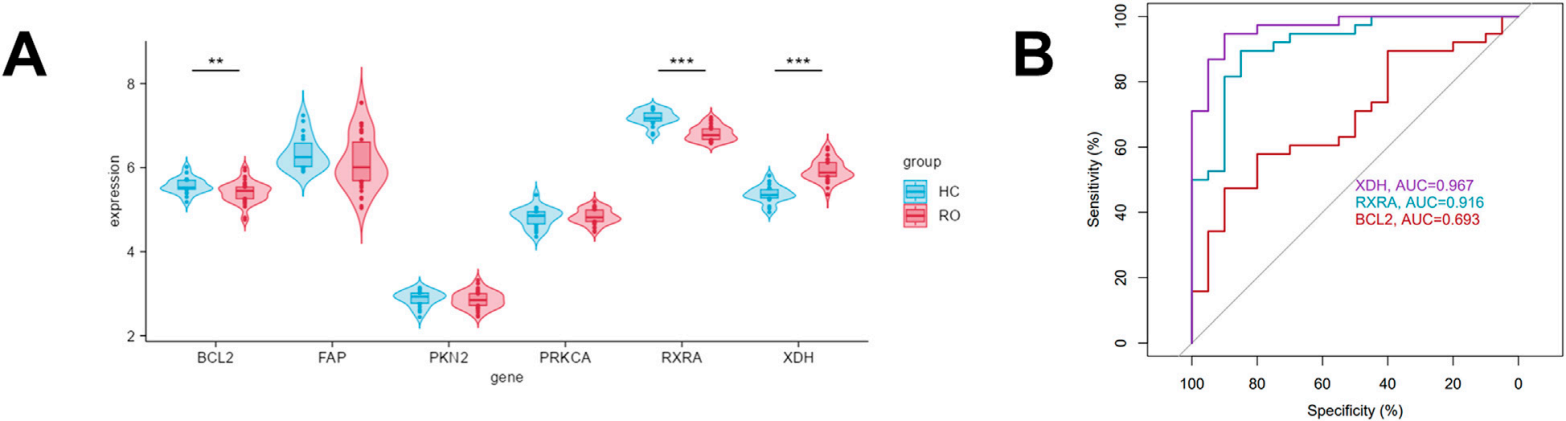
Validation of the expression and diagnostic efficacy of six core targets in rosacea **(A)** Expression of six key targets in biopsy samples from rosacea lesions and healthy controls (GSE65914). **(B)** Diagnostic effectiveness of the three core targets in distinguishing normal and rosacea samples, assessed via receiver operating characteristic (ROC) curves.

Next, we examined the expression profiles of these six core targets in lesional skin *versus* normal skin of rosacea patients. Analysis of the GEO dataset GSE65941 revealed significantly upregulated XDH expression and downregulated BCL2 and RXRA levels in rosacea lesions compared to normal controls, while the remaining genes showed no significant differences (Figure 4A). To further investigate the clinical relevance of these three differentially expressed genes in rosacea, we constructed receiver operating characteristic (ROC) curves for these hub genes and evaluated their diagnostic potential through area under the curve (AUC) calculations. The results demonstrated significant diagnostic power for XDH (AUC = 0.967) and RXRA (AUC = 0.916), whereas BCL2 showed limited discriminative capacity (AUC = 0.693) (Figure 4B). Notably, these findings highlighted XDH and RXRA as particularly crucial molecular players among the six core targets in the pathogenesis of rosacea.

### 3.5 Analysis of the correlation between core targets and immune cell infiltration

**FIGURE 5:**
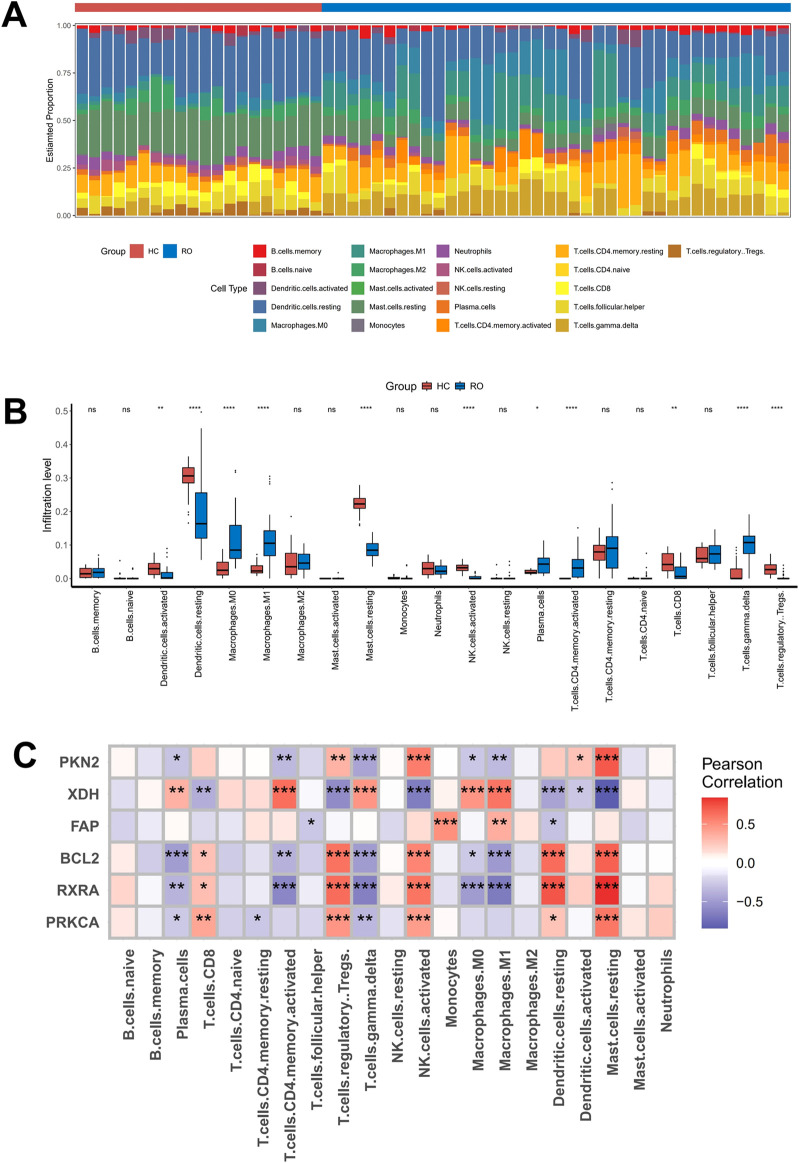
Analysis of the correlation between six core targets and immune cell infiltration **(A,B)** A bar plot comparing the percentage of 21 different types of immune cells found infiltrating in rosacea lesions and normal skin tissues. **(C)** Heat map showing the correlation of core target genes with immune cells.

Previous studies have established a strong association between rosacea and immune dysregulation. To further elucidate the relationship between expression levels of core targets and immune cell infiltration, we performed comparative immune profiling of normal skin *versus* rosacea lesions in the GSE65941 dataset using CIBERSORTx. Our analysis revealed significant alterations in immune cell composition within rosacea lesions compared to healthy controls. Specifically, we observed marked upregulations in macrophages, plasma cells, CD4^+^ memory T cells, and γδ T cells, accompanied by substantial downregulations in dendritic cells, CD8^+^ T cells, and regulatory T cells (Figures 5A,B). Subsequent correlation analysis demonstrated distinct immune interaction patterns of core signature genes. XDH exhibited strong positive correlations with γδ T cells, plasma cells, CD4^+^ memory T cells, and macrophages, while showing significant negative correlations with mast cells and regulatory T cells. Conversely, RXRA displayed inverse association patterns with plasma cells, γδ T cells, CD4^+^ memory T cells, and macrophages (Figure 5C). These findings demonstrated that the pathogenesis of rosacea involved dynamic remodeling of specific immune cell populations, which showed coordinated regulation with the expression levels of key signature genes such as XDH and RXRA. The differential correlation patterns between these molecular targets and immune subsets suggested their potential roles in modulating cutaneous immune responses during disease progression.

### 3.6 Molecular docking of Hup A with core targets

**FIGURE 6:**
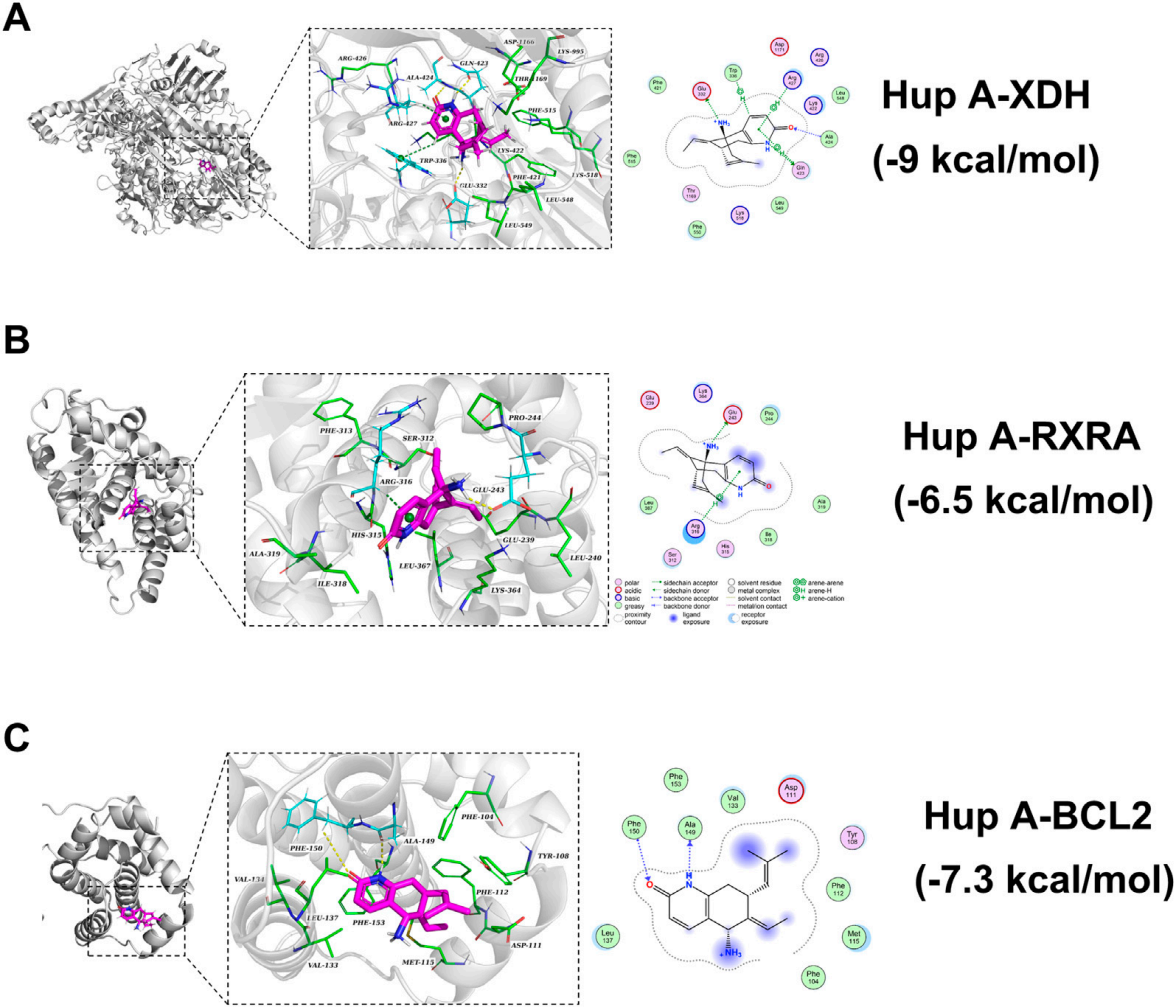
Molecular docking showing the binding affinity of Hup A with six core targets. Binding affinity values are provided to illustrate the strength of the interaction between Hup A and each target. **(A)** Hup A and XDH. **(B)** Hup A and RXRA. **(C)** Hup A and BCL2.

Molecular docking studies were conducted to further investigate the interactions between Hup A and rosacea-associated core proteins, aiming to elucidate the potential mechanism of Hup A in the treatment of rosacea. Machine learning analysis identified six core targets: BCL2, RXRA, PKN2, XDH, PRKCA, and FAP. Among these, differential expression patterns were observed for XDH, RXRA, and BCL2, prompting subsequent molecular docking analysis between Hup A and these three targets. The results demonstrated that all three Hup A-target complexes exhibited docking scores below −6.5 kcal/mol. According to established criteria, lower docking scores indicate stronger binding affinities, with scores <−5.0 kcal/mol suggesting potential binding activity and scores < − 7.0 kcal/mol representing strong binding affinities. Notably, XDH showed the strongest binding affinity with Hup A, achieving a score of −9.0 kcal/mol (Figure 6A). For RXRA and BCL2, the docking scores with Hup A were −6.5 kcal/mol and −7.3 kcal/mol, respectively (Figures 6B,C). The molecular docking results of Hup A with target proteins were shown in Table 2.

**TABLE 2:** Molecular docking results of Hup A with target proteins.

Ligand	Target	Energy (kcal/mol)
Hup A	XDH	−9.0
Hup A	BCL2	−7.3
Hup A	RXRA	−6.5

### 3.7 The results of molecular dynamics simulation

**FIGURE 7:**
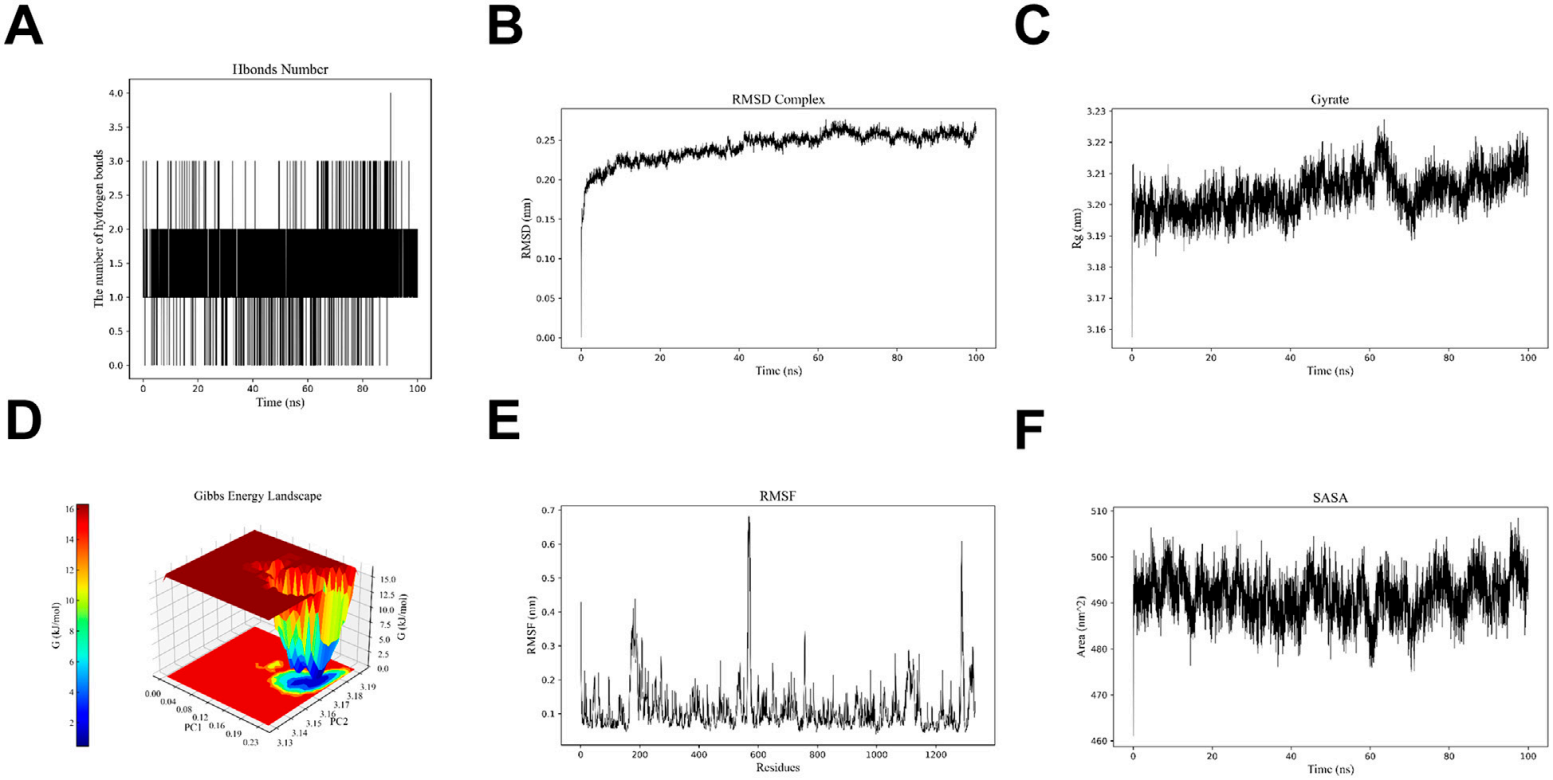
The results of molecular dynamics simulations showed the stability and binding affinity of the XDH - Hup A complex **(A)** Hydrogen bonding pattern analysis results. **(B)** RMSD analysis result **(C)** Rg analysis pattern **(D)** Energy trap analysis result **(E)** RMSF analysis result **(F)** SASA analysis result.

Molecular dynamics analysis of the XDH-Hup A complex was performed based on the lowest binding energy observed between XDH and Hup A, coupled with the significant upregulation of XDH expression in rosacea lesions. Molecular dynamics (MD) simulations were conducted to evaluate the stability of the XDH-Hup A complex through hydrogen bonding analysis (Figure 7A), root-mean-square deviation (RMSD, Figure 7B), and radius of gyration (Rg, Figure 7C), collectively revealing a stable binding conformation. Further characterization via free energy landscape (FEL) analysis (Figure 7D), root-mean-square fluctuation (RMSF, Figure 7E), and solvent-accessible surface area (SASA, Figure 7F) demonstrated a low-energy binding mode, consistent with a thermodynamically stable interaction. These results confirm that XDH and Hup A form a structurally robust complex, aligning with the observed strong binding affinity and supporting XDH as a mechanistically relevant target in Hup A-mediated rosacea intervention.

## 4 Discussion

Rosacea is a recurrent inflammatory skin condition, with a worldwide prevalence exceeding 5% (Gether et al., 2018). Although there are many treatments including topical therapies, systemic treatments, as well as light-based therapy, rosacea cannot be completely cured (Sharma et al., 2022; Hua et al., 2025). Therefore, more effective, and safe therapeutic strategy for rosacea is urgently required. This study integrated network pharmacology, molecular docking, and computational modeling to systematically investigate the potential mechanisms and core targets of Hup A in treating rosacea.

The precise mechanisms underlying rosacea remain elusive, but it is well known that dysregulation of the immune and neurovascular systems has been recognized as playing crucial roles in its pathogenesis (Schwab et al., 2011). Patients with rosacea have an increased risk of depression and anxiety, which may exacerbate flushing and disease progression (Yang et al., 2024; Sinikumpu et al., 2024), underscoring the interplay between neuropsychiatric factors and cutaneous pathophysiology. Hup A was initially identified as an inhibitor of acetylcholinesterase (AChE) based on Chinese databases, and it has been utilized in the treatment of cognitive disorders related to memory deficits, including Alzheimer’s disease and other types of dementia (Damar et al., 2016). By network pharmacology and pathway enrichment analysis, we identified 21 overlapping targets between Hup A and rosacea. KEGG pathway analysis revealed multiple signaling pathways including the MAPK, NF-κB, TNF-a, and PI3K-AKT pathways between Hup A and rosacea (Figure 2). In rosacea, activation of the MAPK signaling cascade drives inflammatory responses via regulation of IL-1β release (Harden et al., 2021). Notably, Isosilybin A has been shown to attenuate rosacea symptoms through MAPK pathway inhibition (Wu et al., 2024), suggesting conserved therapeutic utility of this axis. Similarly, the NF-κB pathway plays a central role in disease progression. For instance, aquaporin 3 (AQP3) overexpression in rosacea activates NF-κB signaling in keratinocytes, promoting chemokine production and inflammatory amplification (Chen et al., 2023). Mechanistically, AQP3-mediated NF-κB activation in keratinocytes synergizes with STAT3 activation in CD4^+^ T cells, creating a feedforward loop that sustains cutaneous inflammation (Chen et al., 2023). This aligns with observations that NF-κB inhibitors, such as metformin and artemisinin derivatives, alleviate rosacea symptoms by disrupting this pathway (Efferth and Oesch, 2021). Our findings suggested Hup A as a multi-target agent capable of concurrently regulating MAPK and NF-κB signaling, thereby addressing the inflammatory core of rosacea. This dual-pathway modulation mirrors the therapeutic strategy of combining psychotropic agents (e.g., paroxetine) with anti-inflammatory drugs in clinical practice, highlighting the potential of Hup A to bridge neuroimmune and inflammatory mechanisms.

Machine learning has become a cornerstone in biomedical research, particularly for identifying disease-critical genes, owing to its capacity to process high-dimensional data and mitigate overfitting by selecting the most predictive features. The robustness of this approach is well-documented. For instance, LASSO regression and SVM-RFE (Support Vector Machine-Recursive Feature Elimination) algorithms have successfully identified psoriasis-specific diagnostic biomarkers by extracting pivotal genes from large-scale datasets (Zhou et al., 2024). In our study, we employed a tripartite computational strategy - LASSO regression, SVM-RFE, and random forest algorithms - to screen feature genes from 21 overlapping targets shared between Hup A and rosacea. The intersection of genes identified by these three algorithms yielded six core candidates: BCL2, RXRA, PKN2, XDH, PRKCA, and FAP. This integrative methodology not only enhances efficiency but also minimizes human bias, offering superior reproducibility and accuracy compared to single-algorithm approaches. By leveraging complementary strengths of LASSO (for regularization), SVM-RFE (for feature ranking), and random forest (for non-linear pattern detection), our framework ensures robust gene prioritization while addressing limitations inherent to individual methods.

To delineate the expression patterns of the six feature genes in the pathogenesis of rosacea, we analyzed GSE65914 datasets comparing lesional and non-lesional skin. Notably, XDH expression was significantly upregulated in rosacea lesions, whereas BCL2 and RXRA were downregulated compared to healthy controls. The other three genes (PKN2, PRKCA, and FAP) showed no differential expression. ROC curve analysis further validated the diagnostic potential of these genes, with XDH exhibiting an AUC of 0.967, RXRA with 0.916, and BCL2 with 0.693, highlighting XDH and RXRA as robust biomarkers for rosacea.

The observed upregulation of XDH aligns with its role in inflammatory responses. Under inflammatory conditions, elevated XDH activity amplifies reactive oxygen species (ROS) production, exacerbating oxidative stress - a key driver of rosacea pathology (Yang et al., 2023; Chaudhary et al., 2023). ROS-activated proinflammatory signaling is critically implicated in immune dysregulation during rosacea progression (Zhang et al., 2022). Conversely, downregulation of RXRA, a ligand-activated transcription factor, may impair its inhibitory effect on the PI3K/AKT pathway (Tang et al., 2018). This pathway comprising phosphatidylinositol 3-kinase (PI3K), protein kinase B (AKT), and mammalian target of rapamycin (mTOR) is pivotal in inflammatory amplification (Hao et al., 2025). In rosacea, LL37 activates mTORC1 via Toll-like receptor 2 (TLR2), establishing a mTORC1-LL37-NF-κB positive feedback loop that drives cytokine/chemokine production and sustains inflammation (Deng et al., 2021). These findings imply XDH and RXRA as central nodes linking oxidative stress and immune dysregulation in rosacea. The therapeutic potential of Hup A may stem from its ability to modulate these targets: suppressing XDH-mediated ROS overproduction and restoring RXRA-dependent PI3K/AKT inhibition. Such dual targeting could disrupt the mTORC1-LL37-NF-κB axis, attenuating inflammatory cascades while addressing oxidative damage - a strategy distinct from conventional monotherapies. Future studies should validate Hup A’s regulatory effects on XDH and RXRA in preclinical models to confirm this hypothesis.

Immune dysregulation is widely recognized as a pivotal driver in rosacea pathogenesis, characterized by the coordinated activation of diverse inflammatory and immune cells (Tu et al., 2024). Our findings revealed that XDH and RXRA exhibit distinct immunomodulatory roles through their interactions with key immune cell populations. Specifically, XDH displayed strong positive correlations with proinflammatory γδ T cells and macrophages, whereas RXRA showed significant negative correlations with these cell types. In rosacea, γδ T cells are markedly expanded and exacerbate cutaneous inflammation via IL-17 production (Zhang Y. et al., 2024). This aligns with evidence that IL-17A-neutralizing antibodies suppress CXCL5/CXCR2 axis activation in murine rosacea models, attenuating both inflammatory and fibrotic responses (Zhang C. et al., 2024). Similarly, macrophages contribute to disease progression through polarized activation. For instance, guanylate-binding protein 5 (GBP5) promotes M1 macrophage polarization via the NF-κB signaling pathway, amplifying skin inflammation in rosacea (Zhou et al., 2023).

To further investigate the binding affinity of Hup A with rosacea-associated targets, computational simulations were employed. Molecular docking revealed robust interactions between Hup A and three core targets, with docking scores below −5 kcal/mol. Among these, the Hup A-XDH complex exhibited the highest stability, consistent with prior findings (Figure 6A). Subsequent molecular dynamics (MD) simulations evaluated the dynamic behavior and structural integrity of the Hup A-XDH complex over time. Key metrics—including root-mean-square deviation (RMSD), radius of gyration (Rg), solvent-accessible surface area (SASA), and hydrogen bond occupancy—confirmed the complex’s structural compactness and stability (Figures 7A–F). These results validate the molecular docking predictions and suggest that Hup A forms a high-affinity, conformationally stable complex with XDH. Mechanistically, this interaction may disrupt XDH-mediated ROS production, thereby modulating immune cell infiltration and attenuating inflammatory cascades in rosacea.

This study integrates network pharmacology, machine learning, and computational modeling to identify six key targets and pathways underlying Hup A’s anti-rosacea effects, with a focus on its binding mechanism to XDH. However, several limitations warrant acknowledgment. First, while computational approaches provide mechanistic hypotheses, *in vitro* and *in vivo* validation is essential to confirm Hup A’s regulatory effects on the NF-κB, MAPK, and PI3K/AKT pathways. Second, the contribution of non-XDH targets (e.g., RXRA, BCL2) to Hup A’s therapeutic efficacy remains to be experimentally disentangled. Future studies should employ CRISPR-based gene editing in keratinocyte or macrophage models to dissect XDH-specific roles, coupled with preclinical trials in rosacea animal models (e.g., LL37-induced mice) to assess translational potential.

## 5 Conclusion

This study established an integrative computational framework - combining network pharmacology, machine learning, and molecular simulations - to elucidate the therapeutic targets and mechanisms underlying Hup A’s anti-rosacea activity. We identified inflammatory signaling pathways, notably the MAPK and NF-κB cascades, as central to Hup A’s action, with XDH, RXRA, and BCL2 emerging as critical molecular targets. Molecular docking and dynamics simulations confirmed Hup A’s robust binding affinities and structural stability with these targets (Figures 6, 7), supporting its capacity to modulate oxidative stress and immune dysregulation in rosacea. These findings underscore the potential of Hup A, a clinically validated neuroprotective agent, as a repurposed multi-target therapy for rosacea. However, translational validation remains imperative: future work must prioritize *in vitro*/*in vivo* validation of Hup A’s effects on the identified pathways and assess its efficacy in preclinical rosacea models. Such efforts could bridge the gap between computational predictions and clinical application, offering a mechanistically grounded alternative to current symptomatic treatments.

## Data Availability

The original contributions presented in the study are included in the article/supplementary material, further inquiries can be directed to the corresponding author.
